# The Susceptibility of Human Melanoma Cells to Infection with the Leningrad-16 Vaccine Strain of Measles Virus

**DOI:** 10.3390/v12020173

**Published:** 2020-02-04

**Authors:** Yulia Ammour, Oxana Ryabaya, Yulia Shchetinina, Elena Prokofeva, Marina Gavrilova, Dmitry Khochenkov, Denis Vorobyev, Evgeny Faizuloev, Igor Shohin, Vitaly V. Zverev, Oxana Svitich, Tatiana Nasedkina

**Affiliations:** 1 I.I. Mechnikov Research Institute for Vaccines and Sera, 105064 Moscow, Russia; yushchetinina@gmail.com (Y.S.); prokofeva_lenka@mail.ru (E.P.); gavrilovamv@gmail.com (M.G.); vorobievdenis@yandex.ru (D.V.); faizuloev.e@gmail.com (E.F.); igorshohin@yandex.ru (I.S.); vitalyzverev@outlook.com (V.V.Z.); svitichoa@yandex.ru (O.S.); 2 N.N. Blokhin National Medical Research Center of Oncology of the Ministry of Health of the Russian Federation, 115478 Moscow, Russia; oxa2601@yandex.ru (O.R.); khochenkov@gmail.com (D.K.); 3 Medicinal Chemistry Center, Togliatti State University, 445020 Togliatti, Russia; 4 I.M. Sechenov First Moscow State Medical University of the Ministry of Health of the Russian Federation, 119146 Moscow, Russia; 5 Institute of biochemical technology and nanotechnology, RUDN University, 117198 Moscow, Russia; 6 Engelhardt Institute of Molecular Biology of the Russian Academy of Sciences, 119991 Moscow, Russia; nased@biochip.ru

**Keywords:** oncolytic viruses, cancer immunotherapy, measles virus

## Abstract

Oncolytic viruses, including live attenuated measles virus (MV) vaccine strains, have recently been shown as promising therapeutic agents against human malignancies. In this study, the oncolytic potential of the attenuated vaccine strain Leningrad-16 (L-16) of MV was evaluated in a panel of human metastatic melanoma cell lines. The L-16 measles virus was shown to replicate within melanoma cells mediating direct cell killing of tumor cells, although all melanoma cell lines varied in regard to their ability to respond to L-16 MV infection, as revealed by the different pattern of the Interferon Stimulated Gene expression, cytokine release and mechanisms of cell death. Furthermore, the statistically significant L-16 measles virus related tumor growth inhibition was demonstrated in a melanoma xenograft model. Therefore, L-16 MV represents an appealing oncolytic platform for target delivery of therapeutic genes along with other attenuated measles virus strains.

## 1. Introduction

Among skin tumors, melanoma occurs in 4% (12.3 % in Russia [1]) of all cases characterized by poor prognosis and low overall survival [2]. Metastatic melanoma is highly aggressive and highly resistant to standard cytostatic chemotherapy, demonstrating moderate sensitivity to immunotherapy and targeted therapy, a 5-year survival of patients with metastatic process ranges between 10% and 15% [2]. The incidence of melanoma is constantly increasing worldwide with an annual rate ranging from 3.0% to 7.0% (4.45% in Russia [1]) and can be considered as one of the highest among all malignant tumors, except lung cancer. From this perspective, new approaches for the therapy of melanoma are highly relevant.

Oncolytic viruses, such as parvovirus H1 [3], reovirus [4], vesicular stomatitis virus [5], vaccinia virus [6], Edmonston derived strains of the measles virus [7] and others have recently been shown as promising therapeutic agents against a broad range of human malignancies. The high efficiency of the oncolytic virotherapy is based on the tumor selectivity and bystander killing effect of oncolytic viruses resulting in antitumor immune response due to the release of tumor antigens coupled with danger-associated and pathogen-associated molecular patterns, DAMPs and PAMPs, and cytokines leading to improving prognosis and overall survival of cancer patients. Rigvir (ECHO-7) was the first oncolytic virus registered and approved for the treatment of melanoma in Latvia in 2004, local treatment of skin and subcutaneous metastases of melanoma, for the prevention of relapse and metastasis after radical surgery in Latvia, Georgia, Armenia and Uzbekistan [8]. In 2015, the engineered oncolytic virus, Talimogene laherparepvec based on a modified herpes simplex virus type 1, was approved for the treatment of unresectable stage IIIb-IV1a melanoma in the U.S., Australia and Europe, while other oncolytic viruses have been investigated as monotherapy or in combination for the treatment of melanoma including herpes simplex virus (HF-10), coxsackieviruses, reoviruses, poxvirus [9] and attenuated measles virus strains of the Edmonsoton vaccine lineage [10]. Current approaches include a combination of oncolytic viruses with radiation therapy, radionuclide therapy, chemotherapy, biologic therapies, and other viruses [11]. Results from phase Ib-II trials combining oncolytic viruses with immune checkpoint inhibitors are promising and have reported overall good treatment tolerance and effect, and phase III trials are ongoing [9].

Previously, the oncolytic properties of the attenuated Russian vaccine strain of mumps virus “Leningrad-3” against human melanoma cells have been shown [12]. The aim of this study was to investigate the oncolytic potential of the attenuated Russian vaccine strain of measles virus “Leningrad-16” (L-16) against human melanoma cell lines and melanoma xenograft model. Despite a different attenuation history compared with the Edmonston B strain, the L-16 measles virus strain has been adapted to preferentially enter cells through the CD46 protein known to be highly expressed on the surface of malignant cells unlike normal cells [13] due to the same amino acid substitution in the 481 position of the hemagglutinin attachment protein. Long passaging adaptation in Japanese quail embryo fibroblast cells of L-16 strain has provided more intensive viral reproduction comparing with Edmonston B and Schwarz strains [14]. In addition, long history of administration of the L-16 attenuated strain as a component of a measles-mumps vaccine in humans in Russia revealed its excellent safety record comparable to the attenuated Edmonston B strain.

## 2. Materials and Methods

### 2.1. Cell Cultures, Virus and Viral Propagation

Human dermal immortalized fibroblast cell line (HDF) and metastatic melanoma cell lines mel Ibr, mel Mtp, mel Il, mel Z, mel Kor derived from patients were obtained from the N.N. Blokhin National Medical Research Center of Oncology (N.N. Blokhin NMRCO). The protocol to establish melanoma cell lines was approved by the N.N. Blokhin NMRCO ethics committee and written informed consent was obtained for all patients. The melanocyte origin of cells was confirmed and the cell lines had been patented previously [15,16].

The Leningrad-16 (L-16) vaccine strain of measles virus (MV), obtained from the collection of the I.I. Mechnikov Research Institute for Vaccines and Sera (titer 5.8–6.0 lgCCID_50_/mL), was propagated in MRC-5 (ATCC, USA) and estimated by the end-point dilution CCID_50_ assays [17] in Vero cells (ATCC).

All cell lines were maintained in T25 and T75 flasks (Corning, USA) in the RPMI 1640 culture medium (Gibco, Thermo Fisher Scientific, USA) supplemented with 10% fetal bovine serum (FBS; HyClone, USA), 1 mM Glutamine (Gibco) and 50 μg/mL Gentamicin Reagent Solution (Gibco) in a humidified atmosphere under 5% CO_2_ at 37 °C. Cultures were passaged once confluent. Cells were split with TrypLE Express (Gibco) containing trypsin, EDTA, and Phenol Red. To assess the cell viability, a cell suspension was supplemented with 0.2% Trypan Blue (Gibco) and cells were counted in a Goryaev chamber.

For viral propagation in melanoma cells, 80%–90% cell monolayers were washed once with 3 mL of Hanks Balanced Salt Solution (HBSS, Gibco) prior to infection. Based on the cell count per flask and virus initial titer, virus stock suspension was diluted in FBS-free culture medium and then used to infect cells at a multiplicity of infection (MOI) of 1.0 or 0.1 infectious units per cell and incubated at 32 ± 1 °C in 5% CO_2_ for 3 h. Mock-infected control cultures were processed in parallel with MV inactivated by exposure to UV light (20.000 μJ/cm^2^, 20 min on ice). Following virus adsorption, the viral inoculum was removed, the cell monolayers were washed three times with HBSS and cultured in RPMI 1640 medium supplemented with 2% FBS in an atmosphere containing 5% CO_2_ at 32 ± 1 °C for 5–7 days. To study the virus accumulation kinetics, supernatants were collected at the indicated time points from infected cell cultures, clarified by centrifugation at 3370× *g* for 10 min in an LMC-3000 centrifuge (Biosan, Latvia) to remove debris and stored at −70 °C until testing.

For gene expression analysis, cells were plated in a 6-well plate (Corning) at 0.5 × 10^6^ cells per well and infected with MV at a MOI of 1.0 or the same dose of UV-inactivated MV. Cells were lysed with 300 μL RLT buffer (RNeasy kit, Qiagen, Germany) per well in duplicates at 24, 48, 72 and 96 h post infection followed by centrifugation for 5 min at 400× *g* (Eppendorf, Germany) stored at –70 °C until use. RNA samples from three independently MV-infected or mock-infected cell cultures were used for each analysis.

### 2.2. Viral and Total RNA Extraction

Viral RNA was isolated from cell culture supernatants using the QIAamp Viral RNA Mini Kit (Qiagen) from 140 μL of the virus-containing supernatant, while total RNA was isolated from cell lysates in RLT buffer using the innuPREP DNA/RNA Mini Kit (Analytikjena, Germany) according to the manufacturer’s spin technology instructions. Purified RNA was eluted twice with 60 μL of RNase-free water and the RNA concentration was determined using the NanoDrop 8000 (Thermo Fisher Scientific): RNA concentration and purity were evaluated by A260 and A260:A280, and A260:A230 ratios. Remaining DNA contaminants were removed by a 30 min digest with 20 U of DNase (Syntol, Russia).

### 2.3. Quantitative Real-Time PCR (qPCR)

Viral RNA quantification was performed as described previously [18]. An amount of 10 μL of RNA was primarily mixed with 2 μL of forward primer at a concentration of 8 ρmol/μL and heated at 65 °C for 5 min. Reverse transcription (RT) was performed on 12 μL of RNA-primer mixture in a final volume of 30 μL with 50 unites of Moloney murine leukemia virus reverse transcriptase (MuLV) (Syntol), 4 units of RNase inhibitor using the 10-fold reaction master mix (Syntol) containing buffer solution, 0,5 mM dNTP and 2,5 mM MgCl_2_. The RT step involved incubation for cDNA synthesis at 42 °C for 30 min and enzyme inactivation by heating at 95 °C for 5 min.

Real-time Taq-Man based PCR was carried out using the 10-fold PCR-RT master mix (Syntol) in a final volume of 25 μL. 5 μL of template cDNA was added to the 20 μL reaction mixture containing forward and reverse primer mix at a final concentration of 10 ρmol per reaction mixture of each primer, TaqMan probe at a final concentration of 5 ρmol per reaction mixture, buffer solution, 0.5 mM dNTP, 2.5 mM MgCl_2_ and 2.5 unites of Hot Start Taq DNA-polymerase. Negative control reaction contained 5 μL of nuclease-free water. Thermal cycling was performed in DT-Prime5 (DNA-Technology, Russia). The cycling conditions included 95 °C for 120 s, 45 cycles of 58 °C for 50 s and 95 °C for 20 s.

Each sample was tested in duplicate. The output of the PCR for each sample was the threshold cycle (C_t_) value measured by the second derivative maximum method of the instrument software. In parallel with samples a 10-fold dilution series of purified reference MV with known titers (expressed in lgCCID_50_/mL) was performed and 5 μL of each standard dilution was run in duplicate to construct a 4-point calibration curve. Titer for the test samples was calculated in CCID_50_/mL relative to reference preparations based on the standard curve and subsequently converted to the lgCCID_50_/mL value.

For gene expression measurement, 1 μg aliquots of each total RNA sample with demonstrated quality were incubated for 1 h at 42 °C with the following components: 1 unit of MuLV reverse transcriptase (Syntol), 5 μM random hexamers or oligo(dT) primers, 1× reaction buffer, 1 mM dNTP, and 20 U RiboLock RNase inhibitor (Thermo Fisher Scientific). The reaction was terminated by heating the mixture for 10 min at 70 °C.

PCRs were performed in a total volume of 25 μL, consisting of 1x SYBR® Green PCR Master Mix (Syntol), 200 nM of reverse and forward gene-specific primers and 10 to 50 ng of cDNA in duplicate reactions. Cycling conditions were as follows: 95 °C for 15 min, followed by 40 cycles of 95 °C for 15 s and 60 °C for 30 s, a melting curve of 10 s at 95 °C, 30 s at 60 °C, heating to 90 °C, and cooling for 30 s at 40 °C, using the DT-Prime5 system. Fluorescence readings were recorded at the last step. A melting curve analysis was performed after amplification to determine the presence of nonspecific amplification products. Dissociation curves for each gene amplified showed only one peak. All the primer pairs used in this study were designed with Primer 3 plus software. Reference genes, *GADPH*, *PGK1* and *ACTβ*, threshold cycle values were used for normalization of the threshold cycle values of the other genes. Specific gene expression, normalized to *GAPDH*, *PGK1* and *ACTb* reference gene average value, was determined using the ΔΔC_T_ mathematical model [19]. The paired two tailed t-test was used to determine statistically significant differences in 2^−ΔΔC^_T_ values between MV-infected and UV-inactivated MV-infected groups. Fold changes were calculated as a difference between 2^−ΔΔC^_T_ values for MV-infected fibroblasts (experiment) versus 2^−ΔΔC^_T_ values for mock-infected fibroblasts (baseline) for each time point after infection. Differences in gene expression with *p* values less than 0.05 were considered statistically significant.

### 2.4. MTT Assay

For each cell line, 1.5 × 10^4^ cells per well were seeded into 96-well culture plates one night before treatment followed by MV-infection using a virus-containing supernatant as described above at various MOI values and incubated in an atmosphere containing 5% CO_2_ at 32 °C for 3–120 h. 3-(4,5-dimethylthiazol-2-yl)-2,5-diphenyltetrazolium bromide (MTT, Promega, USA) reagent was added at 5 μg/mL per well at various time after infection, and cells were then incubated for another 3 h at 32 ± 1 °C in 5% CO_2_. The reaction was stopped by washing cell monolayer and adding 60 μL dimethyl sulfoxide (DMSO, Sigma-Aldrich, Germany) to each well to dissolve the purple formazan precipitates. Plates were shaken at 300 rpm for 10 min to provide for a homogeneous dye distribution. The optical density (OD) was measured at a wavelength of 595 nm using a Zenyth 3100 microplate multimode detector (Anthos, Austria). Cell viability was expressed as a percentage of OD obtained for cell line infected with MV or infected with UV-inactivated MV in an indicated time point relatively to OD obtained for the same cell line at 3 h post infection. The results are described as mean values, which were measured in triplicate and repeated from three independent experiments.

### 2.5. xCELLigence Real-Time Cell Proliferation Measurement

A total 2.0 × 10^3^ melanoma cells per well were seeded in a 16-well E-plate (ACEA Biosciences, Agilent Technologies, USA) and the cell proliferation index was recorded by the xCELLigence system. The experiment was conducted three times and the EC_50_ value representing viral input sufficient for killing 50% of melanoma cells was calculated by the xCELLingence system.

### 2.6. Flow Cytometry

A total 1.0 × 10^5^ melanoma cells were incubated with FITC-conjugated anti-human CD46 antibodies (MEM-258 clone, BioLegend, UK) or Isotype control FITC mouse IgG1 (BioLegend) in PBS containing 1.0 % FBS for extracellular staining at 4 °C. After 30 min at 4 °C, cells were washed twice, resuspended in PBS containing 1.0% FBS and fixed with 1% Paraformaldehyde (PFA, Sigma-Aldrich, USA) for 30 min at 4 °C. Analysis by flow cytometry was performed using the Flow cytometer Beckman Coulter EPICS XL (USA) and the software SYSTEM II (Beckman Coulter, USA).

Annexin-V/PI staining for apoptosis detection was performed using Annexin V-FITC Apoptosis detection Kit (Invitrogen, Thermo Fisher Scientific). A total of 2.0 × 10^5^ cells were harvested, washed in PBS and resuspended in 200 μL Binding Buffer. Cells were then incubated with 5 μL Annexin V-FITC for 10 min at room temperature. Finally, cells were washed in 200 μL Binding Buffer and resuspended in 200 μL Binding Buffer containing 10 μL Propidium Iodide (PI, 20 μg/mL). An analysis by flow cytometry was performed using the Flow cytometer Beckman Coulter EPICS XL and the software SYSTEM II.

### 2.7. ELISA 

The level of TNFα, IL-6 and IL-10 was measured in cell culture supernatants using the Human TNFα, Human IL-6 and Human IL-10 Platinum enzyme-linked immunosorbent assay (ELISA) Kits, respectively, according to the manufacturer’s instructions (Thermo Fisher Scientific). The level of IL-1β and IL-18 was measured in cell culture supernatants using the IL1β-IFA-BEST and IL18-IFA-BEST Kits, respectively, according to the manufacturer’s instructions (Vector-best, Russia).

### 2.8. Agarose Gel Electrophoresis

The PCR products or isolated DNA (innuPREP DNA/RNA Mini Kit, Analytikjena) were resolved electrophoretically in 1%–2% agarose gel in buffer TAE (2 M Tris, 1 M acetic acid, 50 mM EDTA, pH 8.4; Evrogen, Russia). Electrophoresis was carried out at 5–10 V/cm for 1 h. The PCR products or the DNA fragmentation were visualized in UV light at 312 nm and photographed using a Gel Doc XR gel documenting system (Bio-Rad, USA).

### 2.9. Animal Experiments

Five-week-old female athymic Balb/c nu/nu mice (Puschino, Russia) were kept under sterile conditions for SPF-animals. The mice were screened for the absence of viruses, as well as bacterial infections and parasites in accordance with the recommendations of the European Laboratory Animal Science Association. A total of 1.0 × 10^6^ mel Z cells in 300 μL Matrigel (Corning) were injected subcutaneously into the flanks of nude mice. Mice were observed daily and when the xenografts reached 30 mm^3^ in volume, the mice were divided into two groups. One group received three doses of MV L-16 strain in 150 μL intratumorally with a one-week interval, total 1.5 × 10^6^ CCID_50_/mL. The control group received an equivalent amount of RPMI 1640 medium. Mice were euthanized when they lost more than 10% body weight and the average tumor size in the control group reached 450 mm^3^. All animal experiments were performed in accordance with European and Russian national guidelines for animal experimentation and were approved by ethics review committee of the N.N. Blokhin NMRCO (reference number 2017-034, 17.06.2017).

### 2.10. Statistical Analysis

The significance of the difference between experimental and control groups was analyzed using two-tailed Student’s *t*-test. P values were calculated by the unpaired Student’s *t*-test and a value of less than 0.05 was considered statistically significant.

## 3. Results

### 3.1. Susceptibility of Melanoma Cell Lines to the Oncolysis Induced by the Leningrad-16 Vaccine Strain of Measles Virus

Initially, the susceptibility of human melanoma cells to virus-mediated oncolysis was tested in a panel of five cell lines with fibroblast-like morphology derived from patients with disseminated melanoma treated in the N.N. Blokhin NMRCO previously [15,16] and carrying activating mutations in the B-Raf or N-Ras genes participating in the MAPK/ERK signaling pathway (Table 1).

In addition to established melanoma cell lines, immortalized human dermal fibroblasts (HDF) were used as a control cell line to determine virus specificity for melanoma cells.

**Figure 1:**
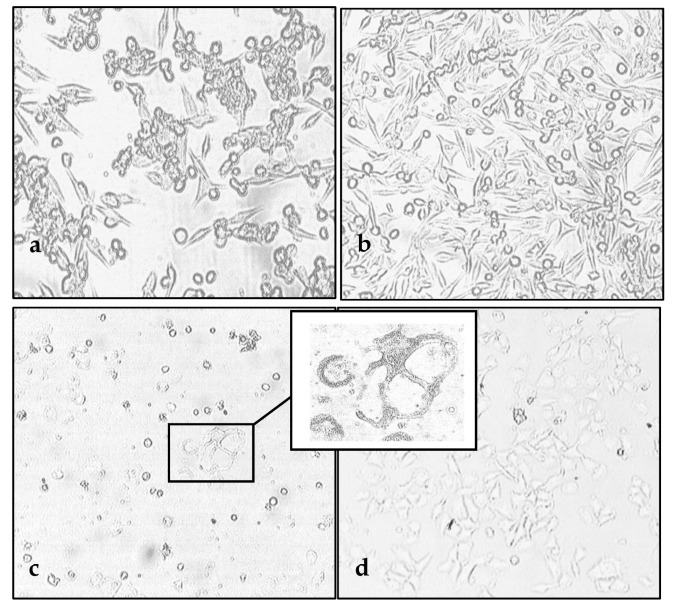

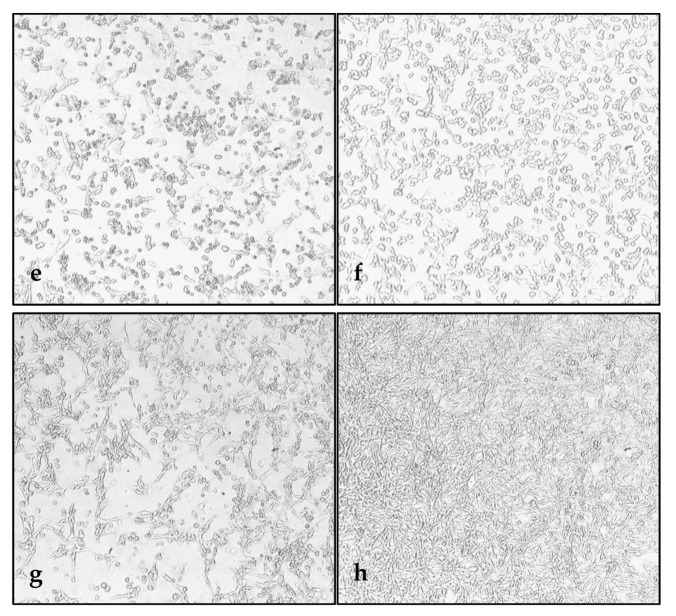
Oncolytic activity of MV L-16 strain against human melanoma cell lines at 72 hours post infection. Mel Il (**a**,**b**, 20×), mel Ibr (**c**,**d**, 10×), mel Mtp (**e**,**f**, 10×) and mel Z (**g**,**h**, 10×) cell lines were infected by MV at a MOI of 1.0 (left column) or mock-infected (right column). The characteristic syncytia formation is highlighted in the footnote.

To analyze the ability of an L-16 strain of measles virus (MV) to eliminate tumor cells following by infection, melanoma cell lines were infected at either a multiplicity of infection (MOI) of 1.0 or 0.1. The cytopathic effects (CPE) of MV in melanoma cells were visible at 48–72 h post infection representing the granulation in cytoplasm and the density loss of cell monolayer followed by the formation of aggregated or giant multinucleated infected cells fused with neighboring cells (syncytia) and sliding off a flask surface progressively. This distinctive MV-induced CPE correlated with MOI and was observed even at a low MOI, becoming more obvious over time (Figure 1).

**Figure 2:**
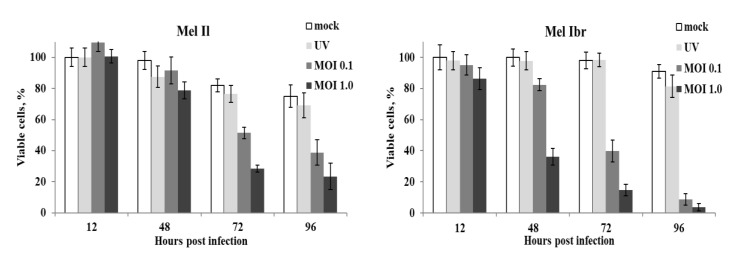

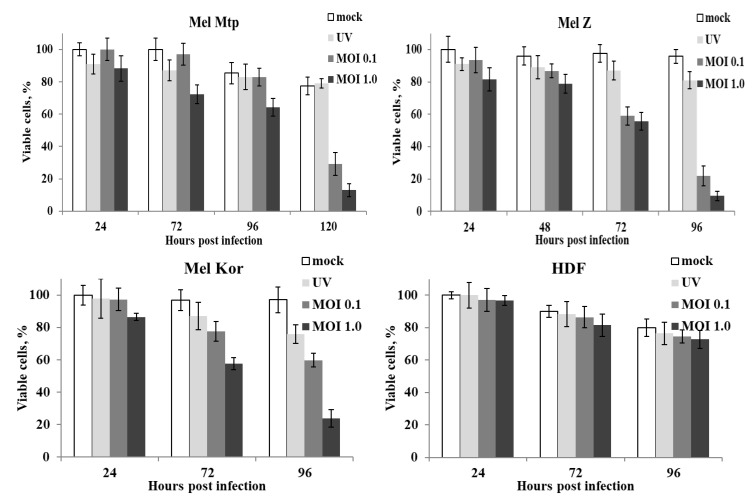
Oncolytic activity of MV strain L-16 against human melanoma cell lines. The viability of each cell line was measured after infection with MV at MOI of 1.0 (black) and 0.1 (gray) or UV-inactivated MV at MOI 1.0 (light gray) using the MTT assay. The data shown are mean results from three separate experiments; error bars indicate standard deviation (SD). The X-axis represents hours post infection, the Y is the percentage of viable cells for each time point relatively to the number of cells at 3 h post infection.

To quantify the virus-induced effect, the percentage of viable cells was estimated at indicated time points during a 120 h MV-infected cell cultivation period by measuring the activity of mitochondrial metabolism in MTT assay (Figure 2). Three out of five melanoma cell lines, mel Il, mel Ibr and mel Z, were eliminated after 96 h post infection even at a low MOI, while two cell lines, mel Mtp and mel Kor, were dying more protractedly. Thus, at 96 h after infection of mel Il, mel Ibr and mel Z cell lines, a proportion of remind viable cells composed 16.2%, 3.7% and 9.5% at a MOI of 1.0 and 26.8%, 8.6% and 21.9% at a MOI of 0.1, respectively, relatively to the corresponding cell line infected with UV-inactivated MV at a MOI of 1.0, while for mel Mtp 64.3% and 82.9%, and mel Kor 23.9% and 59.8% viable cells remained at MOI 1.0 and 0.1, respectively (Figure 2). Interestingly, mel Il, mel Ibr and mel Z carry mutations in *B-Raf* gene, while mel Mtp and mel Kor carry mutations in the *N-Ras* gene (Table 1). Thus, in our study, the viability of the *B-Raf*-mutated cell lines tested decreased after infection with MV at both MOI, 0.1 and 1.0 more significantly compared with *N-Ras*-mutated cell lines. However, the non-malignant HDF cell line turned to be resistant to MV infection and remained viable during 120 h of cultivation (Figure 2).

**Figure 3:**
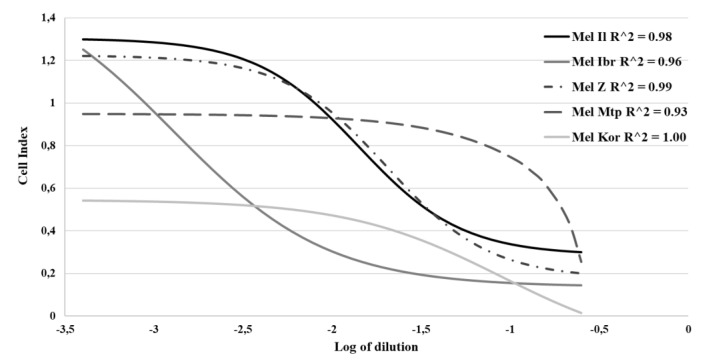
Sigmoidal dose-response for MV-infected melanoma cell lines. The sigmoidal dose-response curves were plotted and ED_50_ dilutions of MV were interpolated using the xCELLigence software for each cell line by the formulae—Y = Bottom + (Top - Bottom)/(1 + 10^(Log EC_50_ − X)). The X axis represents Log of dilution of MV, the Y—Cell Index reflected the number of viable cells.

To calculate the ED_50_ values, real-time proliferation measurement by the xCELLigence instrument was used. Sigmoidal virus dose-response curves were plotted for the log-transformed values within 118–139 h of cultivation with high statistical reliability (R^2^ > 0.9), indicating ED_50_ dilution of input MV as 0.53, 0.008, 0.92, 0.598 and 0.089 CCID_50_/mL for mel Il, mel Ibr, mel Mtp, mel Z and mel Kor cell lines, respectively (Figure 3). Sigmoidal virus dose-response curves had different character depending on cell size and cell proliferation: the amount of cells was higher for mel Il, mel Z and mel Ibr cells comparing with mel Mtp and mel Kor cells at the time of virus inoculation (24 h). Thus, as the initial number of cells was similar for each cell line, the varying values of cell index at the virus inoculation time point indicated different proliferation rate of cell lines. Furthermore, for mel Mtp-infected cells, virus inoculation additionally inhibited their proliferation within the cultivation period. Moreover, cells varied in virus-induced death kinetics: as in MTT-test, mel Ibr cells were the most effectively eliminated cell line; for mel Il and mel Z the death kinetics were similar and had the classical sigmoidal character indicating the ED_50_ 70-fold higher than for mel Ibr cells; for mel Mtp, the ED_50_ dilution of input MV was 1.7-fold higher than for mel Z and mel Il cells, 10-fold and 115-fold higher than for mel Kor and mel Ibr, respectively.

### 3.2. Permissiveness of Human Melanoma Cells to the Leningrad-16 Vaccine Strain of Measles Virus

**Figure 4:**
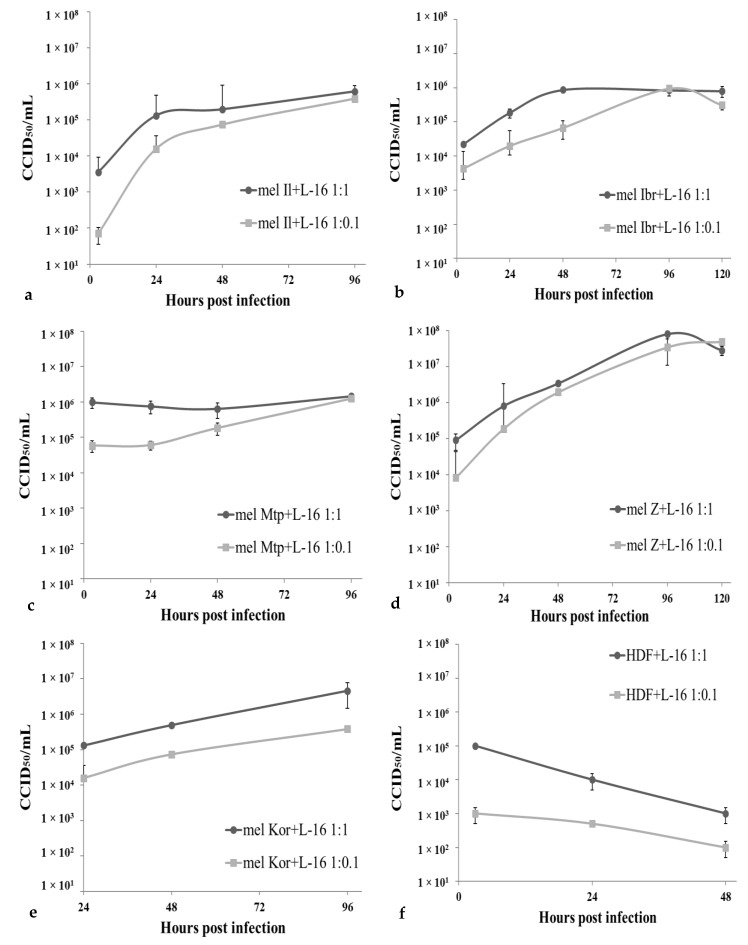
Production of MV strain L-16 in infected melanoma cells and fibroblasts. Melanoma cells, mel Il (**a**), mel Ibr (**b**), mel Mtp (**c**), mel Z (**d**), mel Kor (**e**), or HDF (**f**) cells were infected with MV at a MOI of 1.0 (-○-) or 0.1 (-□-) and virus quantification was determined using the qPCR-RT method in supernatants collected daily from infected cell monolayer. The output of the PCR for each sample was the threshold cycle (C_t_) value. In parallel with samples a 10-fold dilution series of purified reference MV with known titers (expressed in lgCCID_50_/mL) was performed and 5 μL of each standard dilution was run in duplicate to construct a 4-point calibration curve. Titer for the test samples was calculated in CCID_50_/mL relatively to reference preparations based on the standard curve and subsequently converted to the lgCCID_50_/mL value. Means and standard deviations (SD) from three independent experiments are shown.

**Figure 5:**
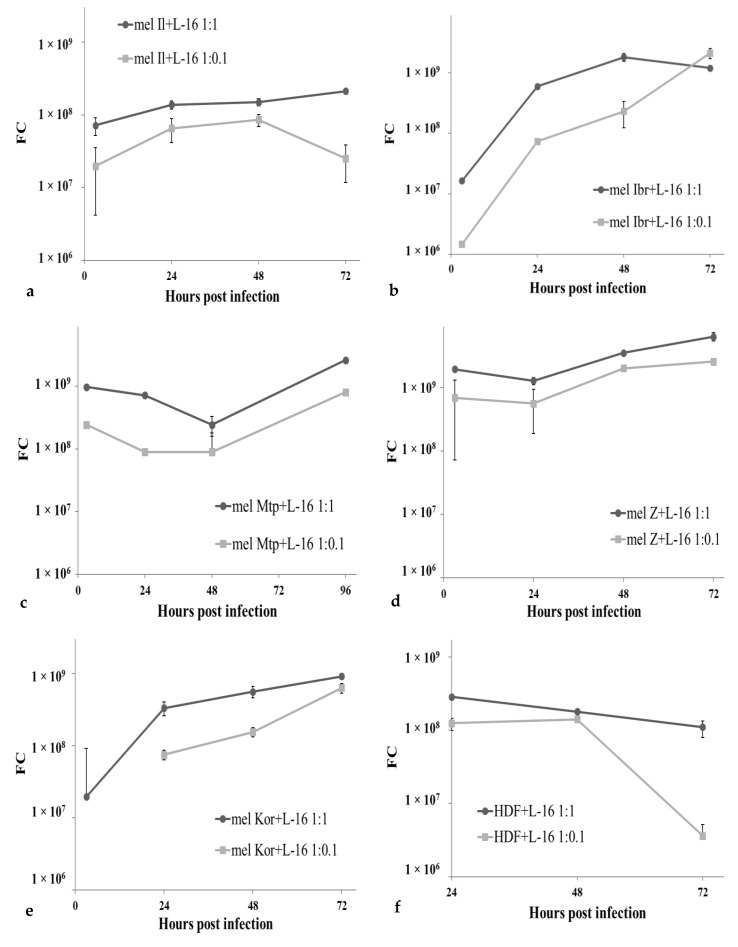
Replication of MV strain L-16 in melanoma cells and fibroblasts. Melanoma cells, mel Il (**a**), mel Ibr (**b**), mel Mtp (**c**), mel Z (**d**), mel Kor (**e**), or HDF (**f**) cells were infected with MV at a MOI of 1.0 (-○-) or 0.1 (-□-) and virus quantification was determined in cell lysates using the qPCR-RT method. The data shown are representative of three separate experiments and expressed in fold change. Fold change—FC, is an average value of threshold cycle (C_t_) for MV intracellular RNA obtained for each sample, normalized to an average C_t_ value for house-keeping genes used: *GAPDH*, *PGK1* and *ACTB*, measured for the same sample in parallel (∆С_t_), and calculated relatively to normalized C_t_ value for the respective mock-infected cell line (∆∆С_t_). The data represents the FC values between the level of MV RNA expression in MV-infected cells (∆∆С_t_(+)) and infected with UV-inactivated MV cells (∆∆С_t_(−)) for each time point, FC = 2^−∆∆Сt(+)^-2^−∆∆Сt(−)^].

To determine whether variations in viral oncolysis observed between different melanoma cells were due to different levels of permissiveness of these cells to the virus infection, the same melanoma cell lines were infected with MV at a MOI of 1.0 or 0.1 again and incubated for 96–120 h. Cell culture supernatants were collected from infected monolayers daily, while cells were washed and lysed. Then, MV replication was measured by real-time RT-PCR (qPCR-RT) both, in supernatants and in cell lysates; obtained results were confirmed by CCID_50_ conventional method (Figure 4 and Figure 5).

Overall, the efficiency of viral RNA replication in all cell lines tested agreed with corresponding cell death kinetics. In supernatants, viral RNA accumulated till 48–96 h post infection almost in all melanoma cell lines, except a high MOI for mel Mtp cells (Figure 4, logarithmic scale). For mel Z cell line, the viral RNA concentration in supernatant reached the highest value among all melanoma cells tested, corresponding to 8 lgCCID_50_/mL at 96 h post infection, increased by 3.0–3.5 log. In mel Il and mel Ibr, 1.5–2.0 and 2.0–2.5 log increase in viral RNA concentration was observed, respectively, in contrast, in mel Mtp and mel Kor, viral titers did not increase by more than 10-fold during 96 h of cultivation.

Whereas, intracellularly, in cell lysates, viral RNA accumulated only from 48 h post infection, except a high MOI for mel Ibr cells (Figure 5, logarithmic scale). That, probably, could be explained by the fastest cell death kinetics among all cell lines and the low number of viable cells remained as the number of cells is known to be the limiting factor for virus replication at high virus input [18]. As for MV RNA accumulation in supernatants, the highest values for intracellular viral RNA concentration were obtained for the mel Z cell line. Interestingly, the lowest viral RNA accumulation was observed for mel Il cell line despite the high cell death effect; furthermore, for the MOI of 0.1 a decrease of viral RNA concentration was observed at 72 h post infection suggesting cellular restriction factors interfering with viral growth. Moreover, a cellular restriction factor should be suggested for mel Mtp cell line functioning until 48 h post infection and lost by 96 h post infection.

Interestingly, despite the similar input of MV while infection, at 3 h post infection the number of viral particles and viral RNA concentration in supernatants and cell lysates, respectively, varied between infected cell lines, thus, MV RNA in mel Il and mel Ibr cells was four times lower comparing with mel Mtp and mel Z cells at 3 h post infection (Figure 4 and Figure 5).

Altogether, the obtained data indicate that all melanoma cell lines tested were permissive for MV infection, although at different levels, while in HDF cells, viral RNA concentration decreased both in supernatants (Figure 4) and cell lysates (Figure 5), indicating that these cells are resistant to MV infection.

### 3.3. Expression of the Measles Virus Receptor, CD46, by the Human Melanoma Cell Lines

**Figure 6:**
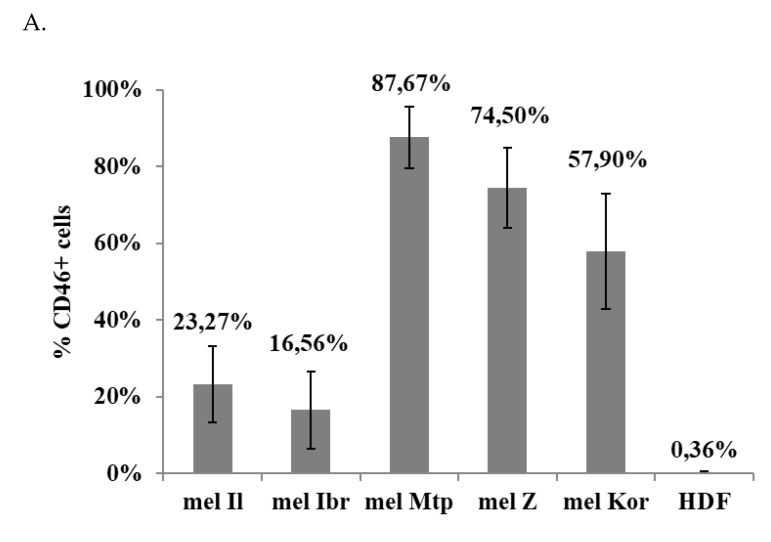

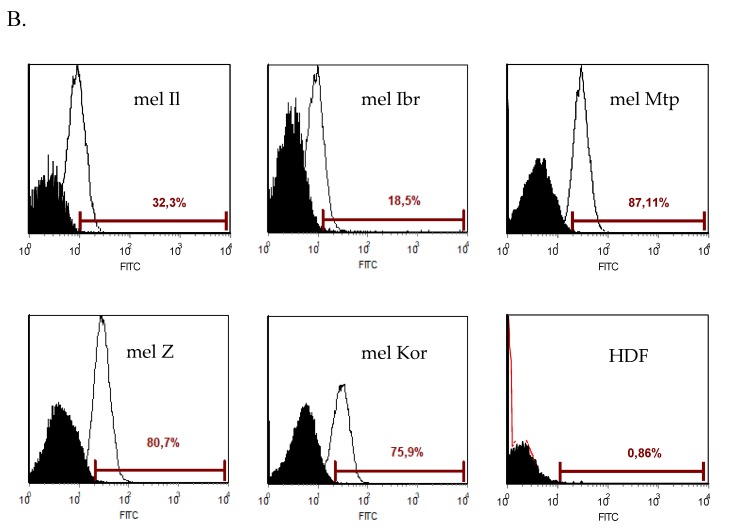
Expression of the CD46 receptor at the surface of melanoma cells and HDF as measured by flow cytometry. A total 1.0 × 10^5^ cells were incubated with a FITC-conjugated anti-human CD46 antibodies in PBS containing 1.0% FBS for extracellular staining. After 30 min, cells were washed twice, resuspended in PBS and fixed with 1% PFA for 30 min. **A**. Plot of fluorescence intensity (relative to isotype) of cells stained with anti-CD46-FITC. The data shown are mean results from three separate experiments; error bars indicate standard deviation of the mean **B**. Representative histograms. The shaded histograms represent isotype control staining; the solid line histograms represent CD46 staining; OX—CD46 (FITC), OY—the number of cells.

To better understand the mechanisms defining the sensitivity of the melanoma cells to MV, the surface expression level of MV receptor was measured. CD46 molecule being a receptor for attenuated strains of MV is known to be upregulated on the surface of tumor cells [13]. Therefore, the expression of CD46 on the surface of melanoma cells and HDF cell line was analyzed (Figure 6).

The expression level of CD46 molecule ranged from 16.56% to 87.67% on the surface of melanoma cells tested, while the HDF cells did not express CD46 (0.36%). Furthermore, viral penetration of the melanoma cell lines increased by increasing the density of receptors on the surface of the cells: thus, for 23.27% and 16.56% of mel Il and mel Ibr cells expressing CD46 receptor, respectively (Figure 6), an initial amount of virus intracellularly composed 7.2 × 10^7^ and 1.6 × 10^6^, respectively, for this cells at 3 h post infection at a MOI of 1.0 (Figure 5), while 87.67% of mel Mtp cells and 74.50 % of mel Z cells expressing CD46 (Figure 6) corresponded to 9.7 × 10^8^ and 1.9 × 10^9^ viral load at a MOI of 1.0 (Figure 5), respectively. However, no significant correlation between different sensitivity of cell lines toward MV oncolysis and expression of CD46 was observed.

**Figure 7:**
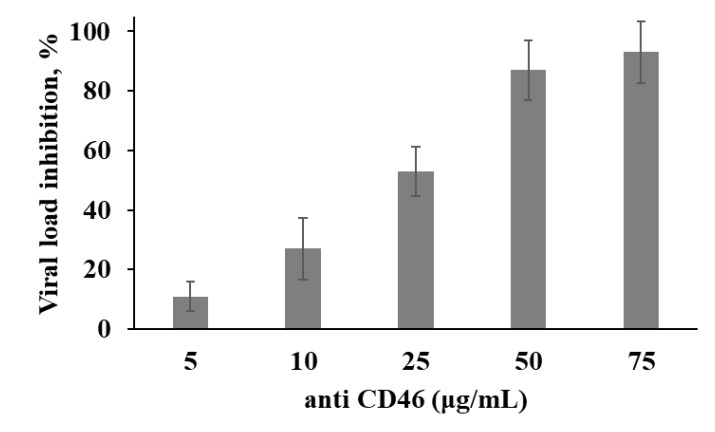
Inhibition of viral load in melanoma cells by mAbs against CD46. Mel Z cell line was incubated with increasing concentrations of mAbs or isotype control mAbs for one hour, washed with PBS and infected with MV at a MOI of 1.0. Viral load was measured in cell lysates by qPCR-RT after 4 h of incubation and the impact of anti-CD46 mAbs was expressed in % relatively to infected cells incubated with isotype control mAbs. The data shown are mean results from three independent experiments; error bars indicate standard deviation.

To confirm the role of CD46 molecule expression on the surface of our melanoma cells in viral oncolysis, the mel Z cells were infected with MV at a MOI of 1.0 again in the presence of anti-CD46 mAbs or isotype control mAbs and viral RNA expression was measured in cell lysates by qPCR-RT after 4 h of incubation (Figure 7). Mel Z cell line was chosen as being sensitive and permissive to MV and expressing high level of CD46 molecules, but in the presence of anti-CD46 mAbs viral yield, was inhibited by approximately 11%–91 % in a mAbs concentration-dependent manner (Figure 7). Thus, these data suggest that CD46 expression could be a key factor of MV selectivity to melanoma cells comparing with non-malignant cells; however, other factors should also be implicated into the different sensitivity of the melanoma cells as it cannot be explained only by different levels of CD46 expression.

### 3.4. Expression of type I IFN Signaling Pathway Genes by Human Melanoma Cells in Response to Measles Virus Infection

**Figure 8:**
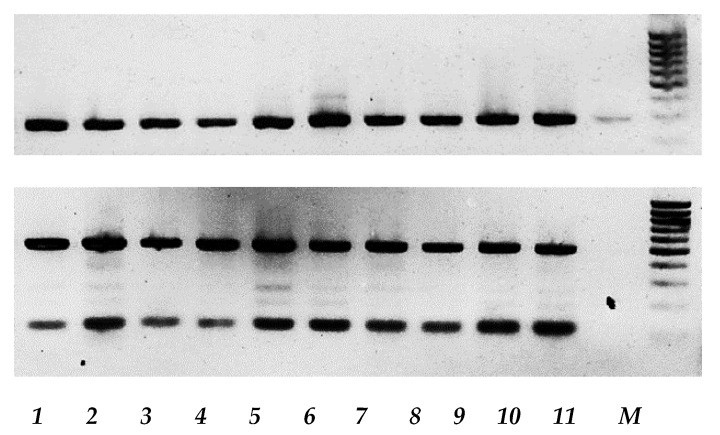
Molecular characterization of DI-RNAs generated by vaccine strain L-16 of MV. cDNA amplified with L-16-specific RT-PCR (upper panel) or DI-RNA-specific RT-PCR (lower panel), and analyzed by agarose gel electrophoresis. The lines correspond to the following cells: 1—mel Il + L-16, 24 h p.i., 2—mel Il + L-16, 48 h p.i., 3—mel Mtp + L-16, 24 h p.i., 4—mel Mtp + L-16, 48 h p.i., 5—mel Ibr + L-16, 24 h p.i., 6—mel Ibr + L-16, 48 h p.i., 7—mel Kor + L-16, 24 h p.i., 8—mel Kor + L-16, 48 h p.i., 9—mel Z + L-16, 24 h p.i., 10—mel Z + L-16, 48 h p.i., 11—input L-16 MV, M—GeneRuler DNA Ladder Mix (Thermo Scientific).

As the previous data suggested other mechanisms in addition to receptor overexpression as possible restriction factors of differential MV infection kinetics of melanoma cells and as other studies previously reported that many cancer cells are defective in type I IFN signaling after viral infection [21], post-entry mechanisms were analyzed. Double-stranded RNA (dsRNA) is a well known trigger of type I IFN signaling, in case of MV infection with L-16 strain, high abundance of defective interfering (DI) particles could play such a role. The presence of DI genomes was confirmed by electrophoresis of PCR products for all cell lines tested (Figure 8) as previously described [22].

Firstly, the expression level of the *DDX58* and the *IFIH1* genes encoding the retinoic acid-inducible gene-1 (RIG-I) and melanoma differentiation-associated 5 (MDA5) helicase proteins, respectively, as intracytoplasmic sensors of viral dsRNA, was measured. A total of 24-48 h after infection, an increase in the gene expression level of these receptors was observed in all cell lines tested, including HDF, indicating that MV was detected by all these cells (Table 2).

Then, we measured the expression level of type I IFN signaling pathway genes encoding regulators of IFN-β transcription—MAVS and IRF-3, IFN-β (*IFNB1* gene) itself together with its receptor subunits—IFNAR1 and IFNAR2, and interferon stimulated genes (ISGs)—*MxA*, *OAS1*, *TNFSF10* (TRAIL), *XAF1*, *EIF2AK2* (PKR), *ISG12/15/6-16*, *IFIT1/2*, *STAT1-3*, *SOCS1/3* encoding proteins involving in the antiviral response of the cell. Although the gene expression levels varied between cell lines after MV infection, certain pattern emerged (Table 2).

The significant increase in MAVS mRNA expression level in cell lines infected by L-16 MV was observed only for mel Ibr cell line (*p* < 0.05), while IRF3 mRNA expression increased almost in all the cell lines tested following by MV infection, except mel Il cells. Three out of five cell lines tested, mel Il, mel Mtp and HDF, expressed IFN-β mRNA at different levels inducing at 24 h post infection and decreasing with time of cultivation, while two cell lines did not express IFN-β mRNA (mel Z cells) or expressed it at a statistically non-significant level (mel Ibr cells, *p* > 0.05). Overall, the pattern of IFN-β mRNA expression did not correlate with sensitivity to MV. However, despite the absence of IFN-β expression induction in mel Ibr and mel Z, this cell line, in turn, induced the expression of certain ISGs—*MxA*, *TNFSF10*, *IFIT2* 24—72 h post infection. It is known that a number of viruses are capable of directly upregulating a subset of ISGs in the absence of type I IFN production [23]. In the mel Mtp cell line, despite a strong induction of IFN-β expression at 24 h post infection, only a small subset of ISGs was overexpressed during the first 24 h post infection correlated with a corresponding peak in IFN-β expression level, such as *OAS1*, *ISG15*, *ISG6-16* (G1P3), *ISG12* and *STAT1* (Table 2), while at 96 h post infection, a substantial induction of *XAF1* (7.8K-fold) and *TNFSF10* genes (TRAIL, 62-fold), two known pro-apoptotic ISGs, was identified (Table 2).

Furthermore, in our study, only HDF and one cell line among melanoma cells tested—mel Il, induced expression of PKR mRNA (*p* < 0.05). The same cell lines induced mRNA expression of IFNAR1 subunit having a higher impact on ISG expression compared with IFNAR2 subunit [24]. Thus, mel Il cell line showed upregulation in response to MV infection for a full spectrum of ISG expression tested at 24 h after infection, although at a low level (Table 2).

Together, our data show surprising diversity among melanoma cells in regard to their ability to express and respond to type I IFN pathway induction indicating the difference in the response to MV infection by all melanoma cell lines tested; however, none of them displayed induction of the total gene set necessary for the inhibition of virus replication.

### 3.5. Release of Inflammatory Cytokines by Human Melanoma Cells After Infection with the Leningrad-16 Vaccine Strain of Measles Virus

**Figure 9:**
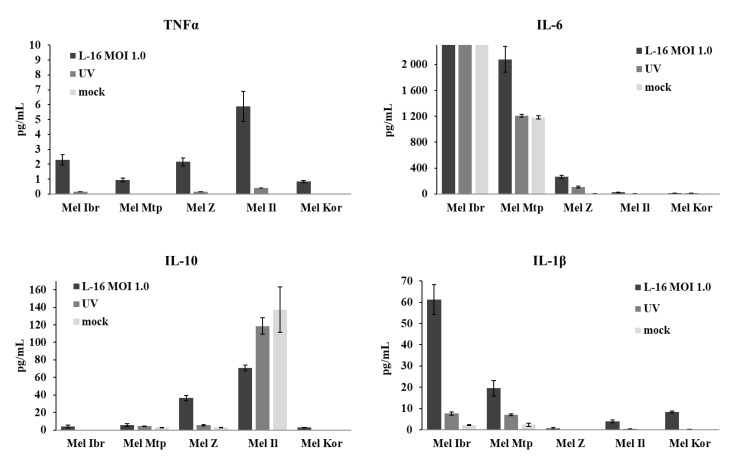
Production of pro- and anti-inflammatory cytokines—TNFα, IL-6, IL-10 and IL-1β release. Cell-free supernatants were collected 48 h after infection with MV or UV-inactivated MV and cytokines levels were determined by ELISA. The data shown are mean results from three independent experiments; error bars indicate standard deviation.

To investigate the ability of melanoma cells to produce an inflammatory response to MV infection, supernatants collected from cells infected with a MOI of 1.0 of MV were analyzed by ELISA for the presence of pro- and anti-inflammatory cytokines—TNFα, IL-1β, IL-6, IL-10 and IL-18 at different time points after infection (Figure 9). The release of cytokines varied between cell lines; however, all cell lines did not express IL-18 at a detectable level (data not shown) but expressed TNFα although at a low level. Uninfected and infected Mel Ibr cells overexpressed pro-inflammatory cytokine IL-6 higher than the detectable level. Mel Mtp cells also expressed Il-6 and increased its expression after MV infection. However, IL-6 can act also as anti-inflammatory agent interacting with TNFα and could potentially favor tumor progression. Both cell lines, mel Ibr and mel Mtp, also expressed the inflammasome formation mediator, IL-1β, at high level comparing with mel Kor and mel Il cell lines, while for mel Z cells the IL-1β expression was not detected. Infection with UV-inactivated MV also induced IL-1β release, although at a lower level comparing with intact MV infection. The separate release of IL-1β without IL-18 cytokine could indicate a non-canonical caspase activation distinct of caspase-1 activation. For two cell lines, mel Il and mel Z, significant expression of anti-inflammatory cytokine IL-10, known among all by regulating the NF-κB activity and Janus kinase-signal transducers and activators of the transcription (JAK-STAT) signaling pathway, was detected. However, in the mel Il cell line, the MV infection decreased release of IL-10 comparing with uninfected or infected with UV-inactivated MV cells. Thus, all human melanoma cells tested responded to the MV infection; however, as for the relative gene expression, no common pattern of cytokine expression was revealed.

### 3.6. Enhancement of Human Melanoma Cell Death Followed by Measles Virus Infection

Previous reports indicated that for some cells, the effect of Ras activation on viral cytotoxicity might be mediated by sensitising the cells to virally induced apoptosis, rather than determining their ability to support viral replication [25]. Other reports showed that cell fusion followed by lysis of cancer cells is a mechanism by which cancer cells are killed by MV [26,27].

**Figure 10:**
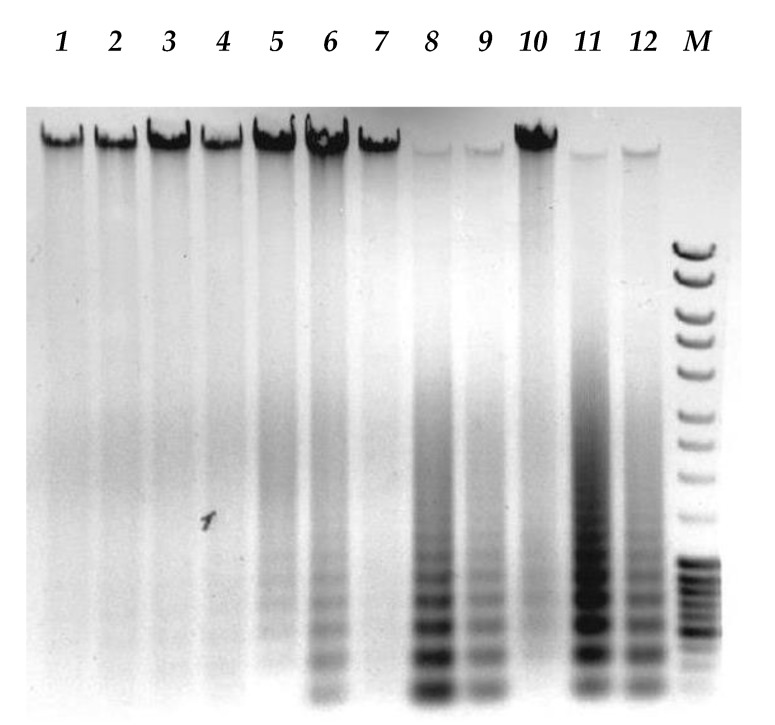
Melanoma cell DNA agarose gel electrophoresis after infection with MV strain L-16. Lines correspond to the following cells: 1—HDF + L-16, 24 h p.i., 2—mel Il + L-16, 24 h p.i., 3—mel Z + L-16, 24 h p.i., 4—HDF + L-16, 48 h p.i., 5—mel Il + L-16, 48 h p.i., 6—mel Z + L-16, 48 h p.i., 7—HDF + L-16, 72 h p.i., 8—mel Il + L-16, 72 h p.i., 9—mel Z + L-16, 72 h p.i., 10—HDF + L-16, 96 h p.i., 11—mel Il + L-16, 96 h p.i., 12—mel Z + L-16, 96 h p.i., M—GeneRuler DNA Ladder Mix (Thermo Scientific).

To understand how MV infection induces cell killing, we examined the effect of MV infection on apoptosis as almost all proteins encoded by the ISG expression tested, except *ISG6-16*, implicated in apoptotic cell death. However, induction of apoptotic cell death was confirmed by characteristic DNA fragmentation in electrophoresis only for mel Il and mel Z infected cells (Figure 10), but not for HDF, mel Ibr and mel Mtp infected cell lines within 96 h post infection.

Mel Mtp and mel Ibr melanoma cells were stained with Annexin V/PI and analysed by flow cytometry in time-dependent maner. The rate of apoptotic cells under MV infection varied between cell lines.

**Figure 11:**
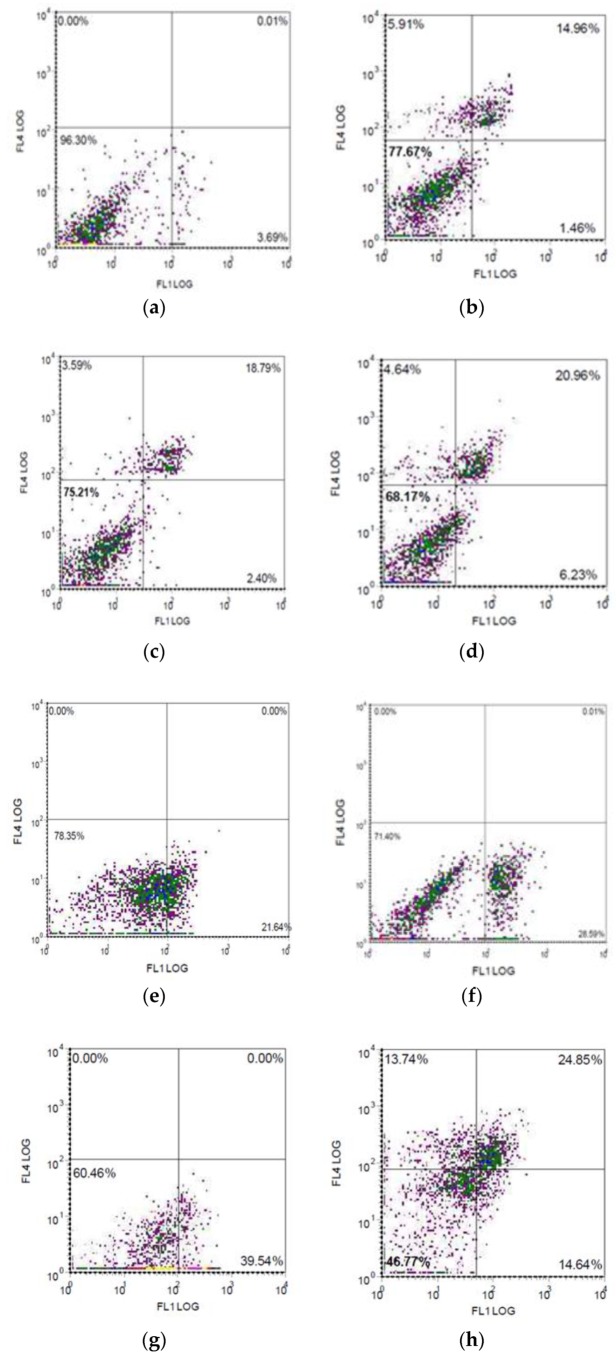
Cytometric analysis of apoptosis in infected melanoma cells stained with Annexin V/PI and analysed by flow cytometry. **a**. Uninfected mel Mtp cell line at 48 h incubation; **b**. MV infected mel Mtp cell line at 24 h post infection; **c**. MV-infected mel Mtp cell line at 48 h post infection; **d**. MV infected mel Mtp cell line at 72 h post infection. **e**. uninfected mel Z cell line at 72 h incubation; **f**. MV infected mel Z cell line at 72 h post infection; **g**. mel Ibr cell line at 72 h incubation; **h**. MV infected mel Ibr cell line at 72 h post infection. ОХ—Annexin V (FITC), OY—PI.

A proportion of Annexin V^+^/PI^-^ cells (suggesting early apoptosis) composed about 1.46, 2.40 and 6.23% at 24, 48 and 72 h post MV infection of mel Mtp cell lines, respectively, and 14.64% at 72 h post MV infection of mel Ibr cells, and was not significant compared with corresponding uninfected cells (Figure 11). While a proportion of AnnexinV^+^/PI^+^ (suggesting late apoptosis) MV infected mel Mtp cells composed 14.96%, 18.79% and 20.96% at 24, 48 and 72 h post infection, respectively, and 24.85% of MV infected mel Ibr cells at 72 h post infection. The lack of early apoptotic cells coupled with the lack of characteristic DNA fragmentation suggests that cells died by a non-canonical pathway orchestrated by membrane integrity lost and phosphatidylserine migration bound with Annexin V coupled with the inflammasome formation resulted in the IL-1β expression. In contrast, for mel Z cells, a proportion of Annexin V+/PI- cells composed about 28.59% at 72 h post MV infection, while Annexin V+/PI+ cells were not detected (Figure 11). These data coupled with the DNA fragmentation detected by electrophoresis and the lack of IL-1β expression could indicate an apoptotic mechanism of cell death. Together, it was shown that melanoma cell lines tested prefer different ways to die.

### 3.7. Potent Antitumor Activity Induced by the Leningrad-16 Vaccine Strain of Measles Virus in Mouse Xenografts

**Figure 12:**
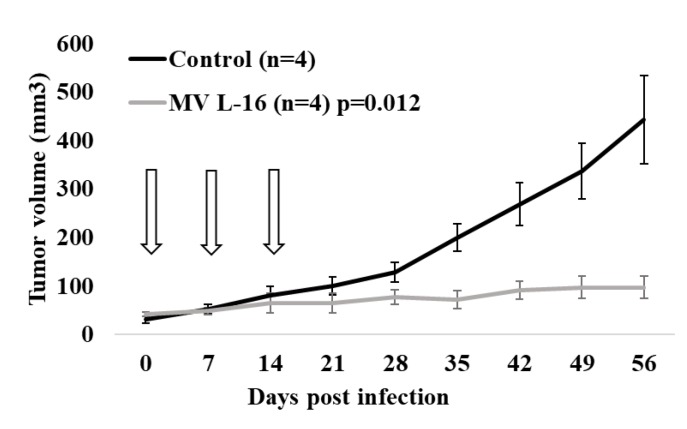
Oncolytic activity of MV strain L-16 against mel Z xenograft tumors in vivo. Mean tumor volume in Balb/c nu/nu mice after three doses of MV L-16 intertumoral injection (n = 4, light grey line) or culture medium (*n* = 4, dark line). The error bars indicate the standard deviation of the mean for each group of animals.

To investigate the antineoplastic efficacy and the therapeutic potential of MV treatment the metastatic mel Z cell line, as sensitive and permissive cell line to MV infection in vitro, was chosen for xenograft development in Balb/c nu/nu mice. Three doses of the MV L-16 strain (1.5 × 10^6^ CCID_50_/mL) were injected intratumorally into established mel Z xenograft tumors by reaching 32 ± 9 mm^3^ in volume on day 14 after implantation. Three doses were injected with a one-week interval. A volumetric analysis of the tumors showed a difference in mean tumor volume between the control group (treated with an equal dose of culture medium) and the MV-treated group (Figure 12). Fifty-six days after treatment, the animals in the control group had a mean tumor volume of 443 mm^3^, whereas the mean tumor volume in the MV-infected animals was 97.25 mm^3^ (*p* = 0.012). Thus, tumor growth inhibition index composed 78.05%. Furthermore, throughout the observation period, the animals in both groups, MV-treated and untreated, survived. After the mice were euthanized, tumors and organs, such as liver, lung, brain, kidney and spleen, were collected and tested for the presence of MV RNA by the qPCR-RT. The traces of MV RNA were detected in tumors, while in the organs, the viral load was below the detectable level.

## 4. Discussion

Melanoma accounts for only 4% of all cancers but is the leading cause of skin cancer death due to its high metastatic potential. While the clinical landscape for melanoma is evolving rapidly, a lack of response to therapies, as well as resistance to therapy remain critical obstacles for treatment of this disease [28].

Oncolytic viruses including live attenuated MV vaccine strains have recently been shown as promising therapeutic agents against human malignant cells. Previous studies using the Edmonston B derived strains of MV have evidenced clinical benefits for the treatment of ovarian cancer, glioblastoma, disseminated multiple myeloma, mesothelioma, breast cancer, osteosarcoma and melanoma [7].

In this study, our attention was focused on the Russian vaccine strain of MV, Leningrad-16 (L-16), carrying the same mutation in the H gene as Edmonston B MV strain leading to preferential attachment to the CD46 receptor despite the different histories of cell culture adaptation of wild types. We demonstrated that the disseminated melanoma cell lines carrying marker mutations in the *B-Raf* or *N-Ras* genes hyperactivating the MAPK/ERK signaling pathway are susceptible to productive infection by MV. Both pre- and post-entry events in melanoma cells in response to viral infection with MV strain L-16 were studied in an attempt to reveal potential mechanisms determining the cell sensitivity to MV.

The main criteria for the safety of oncolytic viruses, including MV, are selectivity of infection and killing toward cancer cells with a minimal effect on normal cells. Thus, such selectivity is possible based on the receptor tropism of oncolytic viruses, as in case of MV overexpression of the CD46 molecules in tumor cells [13], or tumor-related abnormalities in the regulation of mRNA translation suppressing IFN-induced inhibition of cell proliferation and apoptotic signaling, however, also facilitating selective replication of viruses in tumor cells [21]. All cell lines tested expressed CD46 at different levels, thereby accounting for the oncolytic effect observed in most of them. However, we observed limited cell killing with the mel Mtp cell line despite their high CD46 expression level. Hence, we believe that CD46 receptor expression is an important but not essential mechanism and other factors should contribute to the varied effects observed.

There is now evidence that the anti-viral state of the cells likely plays an important role in susceptibility to MV [29]. Indeed, the presence of an intact type I interferon-response pathway in patient tumor samples was shown to correlate strongly with the suppression of MV replication in these cells: the IFN I gene expression level, indicating that the healthy cells and primary tumor cells resistant to virus-mediated oncolysis retained functional antiviral type-I IFN pathways impaired replication of MV, while sensitive cells were defective in these pathways [30,31]. However, other authors reported that melanoma cells retained the ability to release IFN in response to MV infection and were not detrimental to viral killing [10]. In this study analyzing gene expression in response to MV infection in melanoma cells, we also demonstrated that despite the induction of IFN-β gene expression in some cells and interferon-stimulated genes (ISGs), melanoma cell lines were permissive for MV showing varying degrees of sensitivity. For sensitive cell lines, a characteristic cytopathic effect was observed on days 2–3 after infection. Nevertheless, this was accompanied by significant production of defective interfering (DI) genomes represented by short dsRNA sequences, which, in turn, are ligands for intracytoplasmic helicase RIG-I-like receptors. However, DI genomes need a virus as a carrier to reach the cell cytoplasm. Indeed, 24–48 h after infection, an increase in the gene expression level of these receptors was observed, correlating with the IFN-β expression 24 h after infection. As already mentioned, previous authors suggested that most of mesothelioma patient cells were potentially sensitive to MV oncolytic activity due to the defects in the intracellular innate antiviral response by measuring the mRNA expression level of *MxA* gene [28]. We extended a panel of ISG mRNA expression level by measuring *OAS1*, *TNFSF10* (TRAIL), *XAF1*, *EIF2AK2* (PKR), *ISG12/15/6-16*, *IFIT1/2*, *STAT1-3*, *SOCS1/3* gene expression levels and found that in cells missing the expression of mRNA encoding, some based antiviral proteins other ISGs were expressed, while cells remained sensitive to MV inducing cell death. Overall, our data show that melanoma cells varied with regard to their ability to produce and respond to type I IFN, supporting the key role of the IFN signaling in pathway determining the oncolytic effect of L-16 MV leading to ISG induction resulting in apoptotic cascades. Other authors have recently revealed IFN pathway activation as an essential determinant for efficient oncolytic MV infectivity in human glioblastoma specimens by performing RNA sequencing [32,33].

In most human cells, including the fibroblasts, the expression of both IFN-β and a subset of ISGs was shown to be induced early, whereas the expression of the full spectrum of IFNs and ISGs occurs as a second wave dependent on IFN-β secretion [34,35]. Since the IFN-β gene expression by Mel Ibr and Mel Z was never detected following MV infection, the virus probably directly stimulates ISG transcription, sustaining a hypothesis of an IFN-independent pathway in melanoma cells. Indeed, accumulating evidence has demonstrated that numerous ISGs can be directly upregulated following virus infection independent of IFN signaling, whereas typical ISGs are driven by JAK-STAT signaling, other virally stimulated genes are upregulated through the IRF3 and NF-κB pathways [36], including OAS and MxA [37], or through the BATF2-IRF1 as a potential antiviral host machinery in the absence of IFN signaling [33]. These genes are called virus-stimulated genes (VSGs). Interestingly, VSGs have natural anticancer activities protecting normal tissues to enhance the therapeutic index of the virus [10].

Furthermore, the stability of the IFN-β mRNA transcripts during infection with particular viruses is dependent on protein kinase R (PKR) activity preventing deadenylation of the IFN-β transcript [24]. Interestingly, the L-16 strain of MV has additional mutation in MV C protein comparing with the Edmonston B strain, which normally impairs the production of DI [38], thus, the L-16 producing significant amount of DI should strongly trigger the PKR activation, leading to impaired MV growth. However, MV infection of melanoma cells did not lead to an enhanced PKR mRNA expression. Indeed, it was shown that the activated Ras signaling in malignant cells was subsequently found to inhibit the function of PKR, which normally prevents viral protein translation [39]. Thus, in Ras-activated cells, dysfunctional PKR signaling allows virus replication to proceed and cell death ensues. The difference in response toward oncolytic effect induced by MV infection between *B-Raf* mutated and *N-Ras* mutated cells may result from this mechanism. Other groups also analyzed PKR antiviral activity in human lung adenocarcinoma cell lines to the Edmonston B strain of MV and suggested that the PKR antiviral activity plays a role in the limited infection of the resistant tumor cell line by limiting the host transcriptional activity [29].

Thus, the combined low levels or lack of IFN-β and PKR refractory to stimulation, together with the separate expression of ISGs, notably OAS and MxA, could create an environment favorable to MV replication acting as pro-apoptotic and anti-proliferative signals, mediating the death of melanoma cells comparing with normal cells, although the cell death mechanism depended on the cell line tested. Thus, Mel Il and mel Z cell lines were undergoing the characteristic apoptotic DNA fragmentation, confirmed by Annexin V staining, while mel Ibr and mel Mtp melanoma cells may have defective apoptotic pathways and were killed by an inflammatory cell death mechanism leading to the production of a mature pro-inflammatory cytokine IL-1β via the caspase-1-independent pathway disregarding their ability to induce IFN-β expression. It is probable that during viral infection of melanoma cells, delaying apoptosis through autophagy induction would be a viable cellular strategy to protect surrounding uninfected cells from viral infection [40]. Inflammatory melanoma cell death within the tumor microenvironment would lead to innate and adaptive immune activation, limiting MV spread. However, the presence of the virus together with the release of tumor antigens, DAMPs and PAMPs, may overcome immunosuppression in the tumor microenvironment and promote antitumor immunity.

The efficacy and the safety of the L-16 MV were demonstrated in vivo. In a subcutaneous human melanoma xenograft model, the L-16 strain of MV led to the tumor growth inhibition after three injections into established tumors compared with the control group.

Therefore, our obtained data indicating the effective elimination of melanoma cells both in vitro and in vivo, could contribute to the development of approaches for the treatment of metastatic melanoma based on the L-16 strain of oncolytic MV as a platform for further genetical manipulation and could complement the currently available approaches to drug therapy due to the selectivity of viral infection and spreading in tumor tissue with minimal toxic effects on surrounding normal cells.

## Figures and Tables

**Table 1:** Mutations in melanoma cell lines used.

Cell Line	Genome Mutations Determined by NGS [20]	Corresponding Protein Mutations
mel Il	BRAF c.1798_1799delGTinsAA	p.V600K
	BRAF c.1165C > T	p.R389C
	TP53 c.326T > C	p.F109S
mel Ibr	BRAF c.1799T > A	p.V600E
mel Mtp	NRAS c.182A > G	p.Q61R
mel Z	BRAF c.1799T > A	p.V600E
	PDGFRA c.1432T > C	p.S478P
mel Kor	NRAS c.182A > G	p.Q61R

**Table 2:** Type I IFN and Interferon-stimulated genes (ISG) expression at 24, 48 and 72/96 h after infection with MV (MOI = 1.0).

Gene	Fold change														
	mel Il+L-16 vs mel Il			mel Ibr+L-16 vs mel Ibr			mel Mtp+L-16 vs mel Mtp			mel Z+L-16 vs mel Z			HDF+L-16 vs HDF		
	24	48	72	24	48	72	24	48	96	24	48	72	24	48	72
***Intracytoplasmic sensors of viral dsRNA***															
DDX58	78.84	73.26	42.71	21.74	127.59	3.45	922.92	406.33	116.29	30.92	63.05	184.87	41.22	2.55	−0.50
IFIH1	68.51	25.57	13.98	24.49	80.11	3.71	477.59	519.54	548.76	14.25	110.16	270.05	24.99	2.10	0.27
***Cell signaling/ Regulation of transcription***															
MAVS	1.23	0.05	0.02	2.86	12.13	1.71	0.46	1.06	0.81	-0.05	0.38	0.51	1.46	0.00	−0.40
IRF3	1.14	0.08	0.01	0.68	5.90	−0.16	5.73	7.48	10.22	1.25	2.63	6.01	3.29	−0.11	−0.78
***Interferon β and its receptors***															
IFN-β	20.39	4.27	1.47	−0.88	1.75	1.05	5792.62	2521.38	1305.15	−0.33	0.27	1.54	17.38	0.14	−0.35
IFNAR1	2.47	0.11	0.02	0.24	1.91	−1.06	0.37	0.72	1.06	0.78	0.45	1.94	9.56	−0.39	−0.38
IFNAR2	3.88	1.14	0.05	0.15	7.02	−0.56	4.86	4.24	3.36	1.35	0.86	1.32	8.85	−0.79	−1.22
***Interferon Stimulated Genes*** ***Cell defense/immune response/Apoptosis***															
OAS1	41.79	17.73	11.32	3.76	6.37	8.68	5802.42	2918.37	1402.09	299	11.96	7.97	1.47	−0.43	−0.13
MxA	36.25	23.65	16.76	9.20	106.77	95.82	1.64	5.63	5.60	2.24	6.80	18.08	1.74	−0.11	−0.48
EIF2AK2	10.46	2.31	1.02	0.42	0.59	1.92	−0.88	0.28	0.61	0.15	0.29	3.13	761.56	547.91	−0.15
TNFSF10	111.91	39.11	7.28	83.86	659.24	280.84	13.50	43.98	62.37	2.14	18.05	51.31	−0.75	0.19	−0.32
XAF1	44.86	12.38	13.19	5.53	12.44	12.37	2.38	3147.85	7813.10	−0.66	2.44	3.45	2.77	0.12	0.09
IFIT2/ISG54	4.66	4.86	0.01	653.00	653.68	212.44	1.10	1.70	1.08	0.07	2.98	7.89	n/a	n/a	n/a
ISG12/IFI27	2.78	10.88	17.97	n/a	n/a	n/a	24.85	186.39	−1.89	n/a	n/a	n/a	n/a	n/a	n/a
ISG15	5.14	4.60	0.22	n/a	n/a	n/a	6985.52	3144.69	255.73	1.04	12.79	26.90	n/a	n/a	n/a
IFI6/G1P3	2.78	5.81	4.33	0.23	1.55	0.70	129.33	5.31	−1.44	1.89	1.82	2.06	n/a	n/a	n/a
***Signal transducers and activators of transcription***															
STAT1	36.25	21.63	11.89	1.30	0.96	−0.08	75.42	96.80	−0.05	0.91	−1.42	8.97	n/a	n/a	n/a
STAT2	3.53	1.24	0.38	0.30	−0.75	0.00	34.65	3.19	−56.21	0.17	1.46	8.40	n/a	n/a	n/a
STAT3	n/a	n/a	n/a	n/a	n/a	n/a	0.87	−0.42	−0.01	1.24	−1.46	−0.08	n/a	n/a	n/a
***Suppressors of cytokine signaling***															
SOCS3	n/a	n/a	n/a	n/a	n/a	n/a	65.53	−39.54	−1000.40	−0.98	0.49	7.92	n/a	n/a	n/a

FC = Fold Change.

FC < −1000, *p* value < 0.001 
 FC < −10, *p* value < 0.005
−1 > FC > −2
0 > FC > −1, *p* value > 0.05
0 < FC < 2.2, *p* value > 0.05
2.2 < FC < 10, *p* value < 0.05
10 < FC < 30, *p* value <0.01
30 < FC < 60
60 < FC < 100, *p* value < 0.005
FC > 100, *p* value < 0.001
The relative expression levels calculated as an average of three independent experiments with *PGK1*, *ACTb* and *GAPDH* as reference genes in each experiment. Average values of threshold cycles (C_t_) for target gene obtained for each sample were normalized to average Ct values for house-keeping genes, *GAPDH*, *PGK1* and *ACTB*, measured for the same sample in parallel (∆С_t_), and the data were calculated relatively to normalized C_t_ values for the mock-infected cells (∆∆С_t_) for each time point. Fold change represents the difference between ∆∆С_t_ values for MV infected and uninfected cells. Negative values indicate downregulation; n/a label indicates that the obtained C_t_ values did not allow to calculate the relative expression level or the estimation was not performed (for HDF cells).
